# What are the vectors in European livestock farms? Case studies in Hungary and Spain

**DOI:** 10.1186/s13071-026-07392-3

**Published:** 2026-05-08

**Authors:** Clarisse Girault, Richard E. L. Paul, Quentin Narpon, Cristina del Alamo, Roland Sipos, Suthat Lhaosudto, Bruno Passet, Eva Krupa, Gérard Duvallet, José Luis Perez Diaz, Sarah I. Bonnet, Sylvie Manguin

**Affiliations:** 1 HSM, University of Montpellier, CNRS, IRD, Montpellier, France; 2 Ecology and Emergence of Arthropod-Borne Pathogens Unit, Institut Pasteur, Université Paris Cité, CNRS UMR 2000, INRAE USC 1510, Paris, France; 3 Escuela Politécnica Superior, Universidad de Alcala, 28801 Alcala de Henares, Spain; 4 Department of Animal Hygiene, Herd Health and Mobile Clinic, University of Veterinary Medicine Budapest, Budapest, 1078 Hungary; 5 Department of Entomology, Faculty of Agriculture, Kasetsart University, Bangkok, 10900 Thailand; 6 Centre d’Écologie Fonctionnelle et Évolutive, Université Montpellier, CNRS, EPHE, IRD, Université Paul Valéry Montpellier 3, 34199 Montpellier, France

**Keywords:** Arthropod vectors, Mosquitoes, Ticks, Stable flies, Livestock, Farms, Europe

## Abstract

**Background:**

Ongoing global changes are strongly impacting the distribution and incidence of vector-borne diseases (VBD) affecting both humans and animals. Livestock production is a cornerstone of the economy and food security of many countries, notably in Europe, and VBD represent a major constraint on its development. Furthermore, domestic animals can serve as reservoirs for zoonotic agents, highlighting the need for a One Health approach to anticipate and control VBD. However, research on livestock-associated vectors in Europe, particularly mosquitoes and stable flies at farm level, remains limited. Although ticks are recognized as the most important vectors in Europe, comparative studies between countries and host animal species are still scarce. On the basis of vector presence, this study assesses the entomological risk for livestock on seven farms located in two European countries, Spain and Hungary, characterized by contrasting climates and husbandry practices.

**Methods:**

During spring 2023 and 2024, as well as autumn 2023, three groups of arthropods, mosquitoes, ticks, and stable flies were collected from seven cattle, sheep, and pig farms in Spain and Hungary. Environmental, climatic, and meteorological data, together with information on management practices and animal characteristics, were collected on-site and obtained from local databases.

**Results:**

A total of 1432 mosquitoes, 345 ticks, and 1266 stable flies were collected and identified to species level, representing 37 species in total: 30 mosquito species, 6 tick species, and 1 stable fly species. Among these, 16 species are recognized vectors of pathogens. Hungary consistently exhibited higher arthropod abundance across all groups. Mosquito diversity was also greater in Hungary, with 21 species dominated by *Aedes vexans* and *Culex pipiens pipiens*, whereas in Spain, 13 species were recorded, mainly *Culex theileri* and *Anopheles atroparvus*. Four tick species were identified in Hungary (*Ixodes ricinus*, *Haemaphysalis concinna*, *Dermacentor marginatus*, *Dermacentor reticulatus*) while two species were collected in Spain (*Hyalomma lusitanicum* and *Rhipicephalus bursa*). The stable flies *Stomoxys calcitrans* was the only species present in Europe and accounted for all specimens collected, 99% of which were found in Hungary. Seasonal patterns showed spring peaks for mosquitoes and stable flies, and summer/autumn peaks for ticks in Hungary.

**Conclusions:**

The originality of this study lies in its multi-vector description of three arthropod communities associated with three livestock species (cattle, sheep, pigs) on farms located in two European countries with contrasting environments and climates. The study demonstrated the coexistence of 16 arthropod species of veterinary and public health relevance in the surveyed farms. Their diversity and abundance were influenced by geographical contrasts between Mediterranean and Central European climates, as well as environmental characteristics, livestock species, and management practices. These findings provide updated information on the diversity of arthropod vectors present on livestock farms, regardless of production type, and highlighted the need for enhanced vector surveillance in livestock systems, which accounts for environmental, farming, and anthropogenic factors. Such efforts are essential to anticipate VBD emergence driven by invasive vectors and circulating pathogens, mitigate impacts on animal health and productivity, and address interconnected risks to both human and animal populations.

**Graphical abstract:**

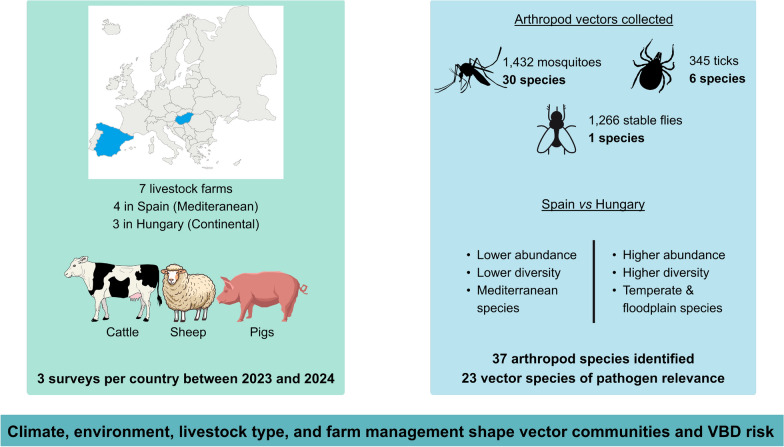

**Supplementary Information:**

The online version contains supplementary material available at 10.1186/s13071-026-07392-3.

## Background

With the global livestock sector generating US$1.27 trillion annually, it accounts for 40% of global agricultural production and supports the livelihoods and food security of approximatively 45 million farmers in developed countries and 1.3 billion people in developing countries [1]. However, this crucial contribution to the global economy, food security, and livelihoods rests on a delicate balance. Animal and human health are intrinsically interconnected and closely to the environmental conditions. In the context of ongoing global changes, numerous challenges are emerging, including the increasing threat of vector-borne diseases (VBDs) [2].

Most studies in Europe have focused on a single vector genus/species and/or pathogen, such as the transmission of bluetongue virus by *Culicoides* spp. In contrast, our approach is both novel and integrative, as it simultaneously documents the diversity and abundance of three groups of arthropod vectors that have not previously been studied together in the context of livestock farming. These include two groups well known for their veterinary importance, ticks and stable flies, and a third group, mosquitoes, which remain largely understudied on farm environment [3–5]. Ticks are among the most important vectors of animal pathogens worldwide, transmitting a broad range of parasites, bacteria, and viruses [6]. Stable flies (*Stomoxys* spp.) are mechanical vectors capable of transmitting pathogens by resuming interrupted blood meals on new hosts [7]. Mosquitoes, widely recognized as the primary vectors of parasites and viruses affecting humans worldwide, also transmit pathogens to animals, including West Nile virus (WNV) and Usutu virus (USUV) [8, 9].

Beyond their impact on animal health, VBDs also pose risks to humans living in proximity to domestic animals, due to the circulation of zoonotic pathogens in animal reservoirs. Approximately 75% of emerging infectious diseases are zoonotic, many vector-borne, and about 25% originate in domestic animals [10, 11]. Surveillance of VBDs in livestock populations may therefore facilitate early detection and anticipation of zoonotic disease emergence [12]. Furthermore, by providing blood meals for hematophagous arthropods, domestic animals can contribute to increased vector population densities [13]. In addition to pathogen transmission, hematophagous arthropods have significant economic consequences for livestock production. Intensive biting activity by stable flies and ticks causes stress, blood loss, and behavioral disturbance in cattle, leading to reduced weight gain and milk production, as well as damage to hides, affecting the leather industry [14, 15]. Nuisance caused by dipteran flies has been reported to reduce milk production in cattle by 20–30% [16].

Vector ecology and distribution are shaped by their host availability and environmental conditions, including temperature, relative humidity (RH), vegetation, and land use. The intensification of human and animal activities, together with socioeconomic and environmental changes associated with ongoing global change, further modifies vector populations. Both abiotic factors (e.g., temperature, rainfall, RH) and biotic factors (e.g., animal abundance, animal size, herbaceous vegetation cover) influence vector densities and activity. Consequently, shifts in vector distributions are altering the risk of VBDs, making the emergence and reemergence of these diseases an increasing threat to both human and animal health [17]. Vector competence refers to the intrinsic ability of an arthropod to acquire, maintain, and transmit a given pathogen; importantly, each arthropod species is capable of transmitting only specific pathogens [18]. Therefore, the identification, distribution, and abundance of arthropod vectors, together with the intensity of host–vector interactions, are key indicators of VBD risk. Although contrasting vector assemblages between Mediterranean and Central Europe regions have been reported, available data remain largely fragmented across vector groups [19, 20].

A simultaneous investigation of three vector communities on farms located in distinct geographical regions, characterized by diverse climates, livestock species, environmental settings, and husbandry practices, is essential to accurately characterize local vector assemblages. Such an approach enables improved assessment of the transmission dynamics of vector-borne pathogens and their potential impact on animal health. This knowledge is critical for designing and implementing effective and context-specific control strategies. In this framework, the present study aims to identify mosquitoes, ticks, and stable flies on cattle, sheep, and pig farms located in selected regions of Spain and Hungary. The objective is to provide an updated, farm-scale description of arthropod communities and the potential presence of vector species under contrasting climatic and farming conditions.

## Methods

### Study areas

**Fig. 1:**
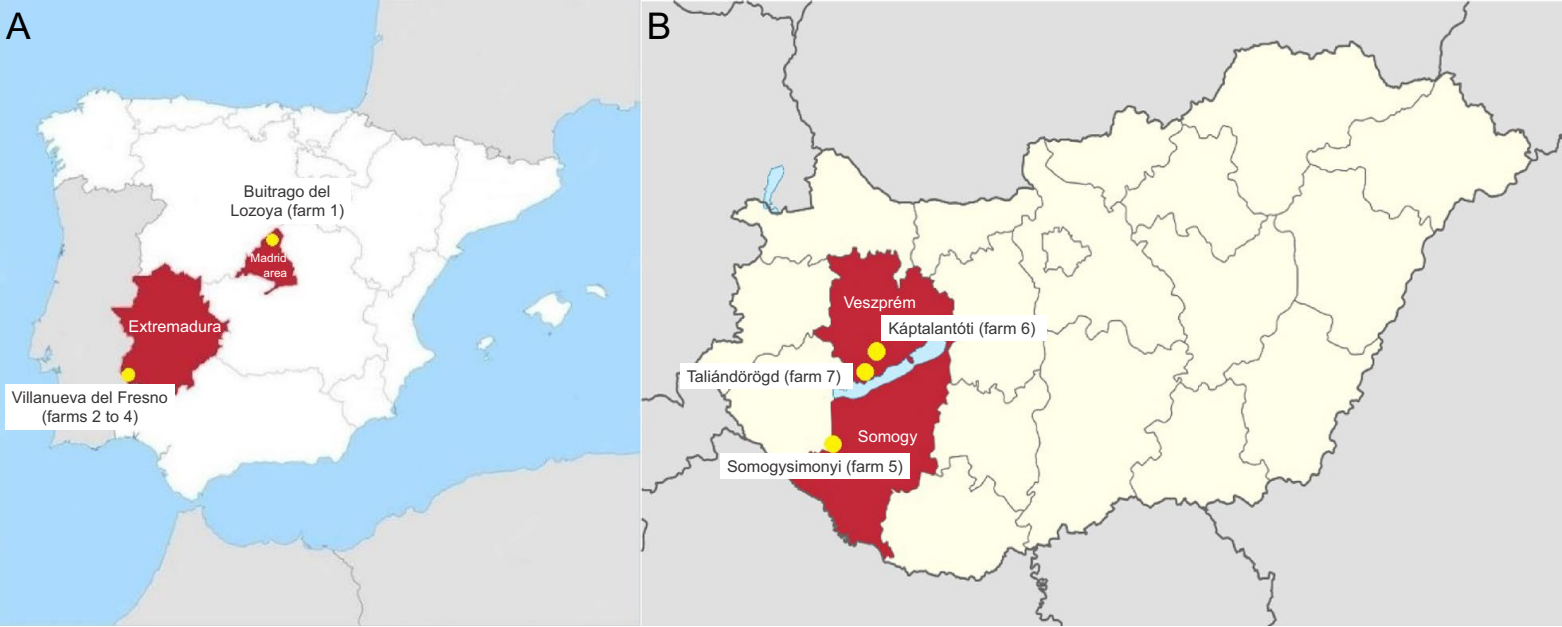
Map of Spain (**A**) and Hungary (**B**) indicating the locations (yellow dots) of the seven farms where mosquitoes, ticks, and stable flies were collected. Farm 1: Casa de Riosequillo (Madrid region, Spain); farm 2: La Bogoña, farm 3: El Carril, and farm 4: La Carballa (Extremadura region, Spain); farm 5: Geum Kft (Somogy county, Hungary); farm 6: Istvándi és Társai Kft, and farm 7: Dörögdi mező Kft (Veszprém county, Hungary)

Mosquito, tick, and stable fly collections were conducted during three surveys in Spain (May 2023, October 2023, and April 2024) and three surveys in Hungary (July 2023, September 2023, and July 2024). These periods correspond to the main activity seasons of arthropod vectors, thereby maximizing capture success and enabling seasonal comparisons across farms and countries [21–23]. In Spain, four farms were selected: Casa de Riosequillo cattle farm in Buitrago del Lozoya, Madrid (farm 1), and three in Villanueva del Fresno community, Extremadura: La Bogoña pig farm (farm 2), El Carril sheep farm (farm 3), and La Carballa, with both sheep and cows (farm 4) (Fig. 1A). In Hungary, three farms were selected: Geum Kft cattle farm in Somogysimonyi (farm 5, Somogy county), and two in Veszprém county, Istvándi és Társai Kft pig farm in Káptalantóti (farm 6) and Dörögdi mező Kft sheep farm in Taliándörögd (farm 7) (Fig. 1B). Oral or written consent was obtained from all farm owners prior to inclusion in the study.

**Fig. 2:**
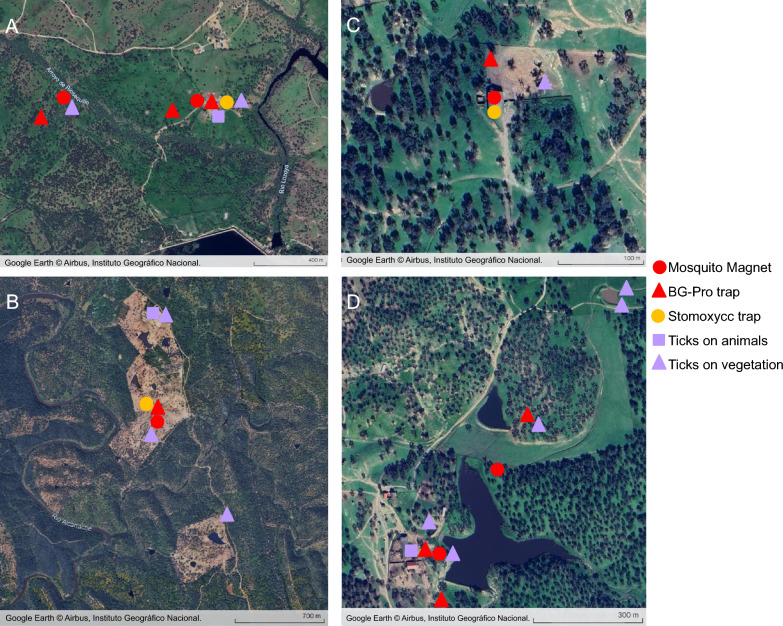
Satellite views from Google Earth of the four livestock farms sampled in Spain: **A** Casa de Riosequillo, Madrid region (farm 1, cattle), **B** La Bogoña (farm 2, pigs), **C** El Carril (farm 3, sheep), and **D** La Carballa (farm 4, cattle and sheep), B–D: Extremadura region

**Fig. 3:**
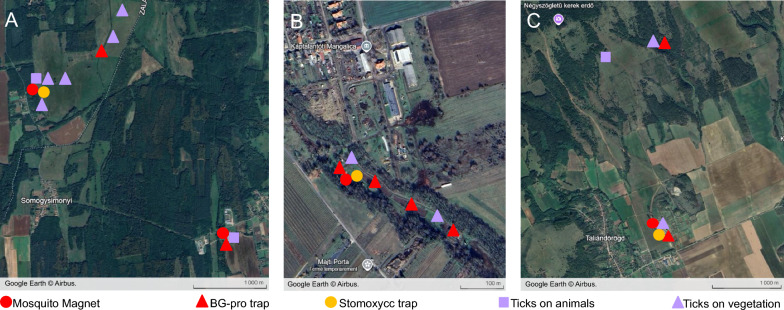
Satellite views from Google Earth of the three livestock farms sampled in Hungary: **A** Geum Kft (farm 5, cattle), **B** Istvándi és Társai Kft (farm 6, pigs), and **C** Dörögdi mező Kft (farm 7, sheep)

Sampling dates and meteorological variables (temperature and humidity), as well as additional farm characteristics including GPS coordinates, altitude, farm size, vegetation type, number and breed of animals, farming practice and reported wild fauna, are presented in Additional file 1. Climatic zones were assigned using Köppen–Geiger climate classification [24]. Animals were kept in pastures or enclosures surrounded by electric or non-electric barbed-wire fencing. The Hungarian Istvándi és Társai Kft pig farm was an exception, as it complied with African swine fever (ASF) regulations requiring a three-level fencing system, including a 2-m-high perimeter fence and an additional electrified fence. Satellite views of each farm showing arthropod collection sites are presented in Figs. 2 and 3.

### Arthropod collections

*Mosquitoes*. The mosquito collections were carried out during three surveys in each country using two types of traps that were simultaneously implemented at each study site to collect adult mosquitoes. The Mosquito Magnet Executive model® (Woodstream Corp, Lititz, PA, USA) (MM), with an estimated coverage area of 2000 m^2^, converts propane into CO_2_, and it was used with Atrakta Lurex® to attract *Aedes albopictus*, and R-Octenol for other mosquito species, in accordance with the manufacturer’s recommendations. The BG-Pro® (Biogents, Regensberg, Germany), generating CO_2_ through a yeast–sugar–water fermentation system maintained at 37 °C, was placed either on the ground or suspended above ground level. Traps were deployed indoors or outdoors (inside or near stables), and close to water bodies and/or livestock whenever possible. According to the size of the farm, up to two traps of each type were installed per farm, with a maximum of four traps per farm, positioned at least 5 m apart. The MM trap was oriented in the opposite direction to the BG trap to minimize potential interference. Collected mosquitoes were preserved in 2–5 mL of RNA*later*™ Stabilization solution (Thermo Fisher Scientific Baltics UAB) for up to 10 days, subsequently rinsed with RNA-free water, and stored at −80°C until further processing.

*Ticks*. Nymph and adult ticks were collected once per survey on each farm on animals and vegetation (pastures, forest and accessible vegetated areas). Between 5 and 10 animals per farm, depending on accessibility, were examined by visual inspection and palpation, and ticks were removed using a tick twister. Vegetation sampling was performed using dragging and/or flagging methods depending on vegetation accessibility. For dragging, a 1 m^2^ white cloth was pulled over vegetation along 10-m transects. For flagging, a 50 × 60 cm white cloth was swept over vegetation for 15 min at each sampling point. The number of collection points (dragging and/or flagging) reflected farm size, with a minimum of four transects and/or flagging points, and up to eight, per farm. The same points and collection times (flagging as dragging) were used once during the three surveys, for each farm in each country. All specimens were also preserved in 2–5 mL of RNA*later*™ for up to ten days, rinsed with RNA-free water and stored at −80 °C until further processing.

*Stable flies*. Stable flies were collected using one StomoxyCC trap (Alcochem Hygiene Company, https://stomoxys.com, Holland) operated for 24 h per farm and per survey, except on cattle farm 5 in Hungary, where two traps were deployed due to the presence of two manure sites. Traps were mounted on iron stakes at approximately 30 cm above ground level, placed near livestock and manure, in open, sunny areas, and surrounded by a protective barrier [25], with the exception of the trap in El Carril, which was set in the stable near sheep and manure. Captured flies were collected in 70% ethanol, transported to the laboratory, transferred to 90% ethanol, and stored at −80 °C until analysis.

### Arthropod identification

Morphological identification was performed under a stereomicroscope. Adult mosquitoes were identified to genus level using the MoskeyTools software and the “Les genres de moustiques d’Europe” database (MIVEGEC, https://mivegec.fr/fr/identiciels) [26]. Ticks and stable flies were identified to species level using taxonomic keys [27, 28].

Female mosquito identification at the species level was performed by amplification and sequencing of DNA barcoding regions. DNA and RNA were extracted from abdomen tissue in a final elution volume of 20 µl using the AllPrep® PowerFecal® Pro DNA/RNA Kit (Qiagen, Hilden, Germany), following the manufacturer’s instructions, with minor modifications: homogenization was performed with the addition of 100 µL phosphate-buffered saline (PBS), and lysis volumes were reduced by half. Extracted RNA and DNA were stored at −80 °C and −20 °C, respectively. For *Culex* and *Aedes* species, a 709 bp fragment of the mitochondrial cytochrome oxidase subunit I (*COI*) gene was amplified by polymerase chain reaction (PCR) following the protocol of Folmer et al. [29], with the following modification: 1 U of GoTaq® Flexi DNA polymerase per reaction (Promega, Madison, USA), 2.5 mM MgCl_2,_ 0.2 µM of each primer, and a final reaction volume of 30 µl. Thermal cycling conditions followed Becker et al. [30], except that an annealing temperature of 50 °C was used during the final 35 cycles. For *Anopheles* and *Culiseta* species, a 350–600 bp fragment encompassing the internal transcribed spacer 2 (ITS2) region was amplified by PCR using the same protocol, except that MgCl_2_ concentration was adjusted to 1.5 mM. Primers described by Collins et al. [31] and PCR conditions followed Garros et al. [32], with the final extension step reduced to 5 min. The obtained amplicons were sent to Genewiz Europe (Leipzig, Germany) for purification and sequencing using the Sanger method. Sequence alignments and species identification were performed using the GenBank database and the BLASTN algorithm (National Center for Biotechnology Information, Bethesda, MD, USA). All female mosquitoes collected in Spain were subjected to species-level identification due to the limited number of specimens. In Hungary, given the large number of collected individuals, a representative subset of female mosquitoes was selected a posteriori for molecular identification on the basis of farms, seasons, livestock types, and trapping methods.

### Statistical analysis

Factors associated with the number of collected mosquitoes were analyzed using generalized linear models with a Poisson regression. The explanatory variables included year, season, country, livestock type, mosquito sex, and trapping method (MM versus BG-Pro). For *Stomoxys*, country, livestock type, season, and year were fitted as factors and the total number of flies as the output variable. Multivariable analyses were performed, with sequential removal of nonsignificant parameters. Final models retained only significant variables. Overdispersion parameters were estimated and applied to account for any over-dispersion in the data that could generate spuriously small *P*-values. Because the data did not have a normal distribution, a Poisson regression was performed with a log link function; Wald statistics, which approximate to a *χ*^2^ distribution, were used to evaluate the association between explanatory factors and outcome variables. The corresponding *t*-values were then used to compare groups within the explanatory factor. Association analyses were performed in Genstat v22 [33]. Tick data were analyzed descriptively, due to heterogeneity in sampling effort and variability in farmer-implemented tick prevention measures among the studied farms, and limited sample sizes in Spain.

## Results

### Mosquito collections and identifications

**Fig. 4:**
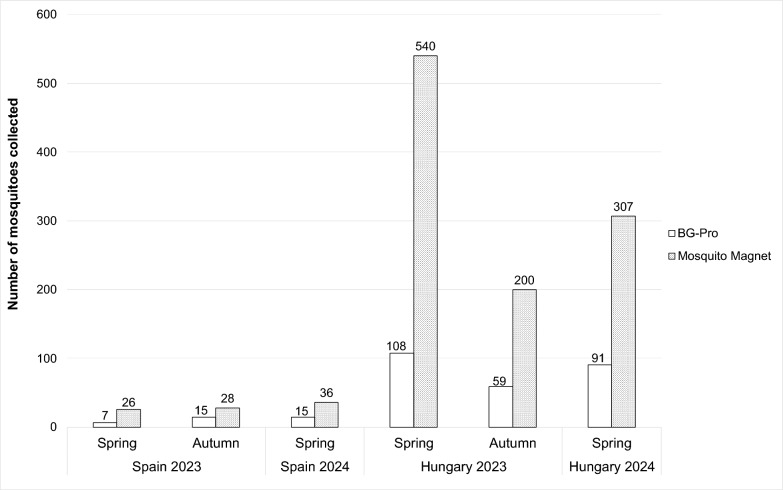
Seasonal and geographical variations in adult mosquito collections performed using BG-Pro and Mosquito Magnet traps in Spain and Hungary in 2023 and 2024. Histogram patterns: open rectangles, BG-Pro; dots, Mosquito Magnet

In total, 127 mosquitoes were captured in Spain (107 females, 18 males, and 2 undetermined specimens), compared with 1305 in Hungary (1275 females, 27 males, and 3 undetermined) (Fig. 4). The number of adult mosquitoes collected in Hungary was therefore approximately tenfold higher than in Spain (*χ*^2^_1_ = 26.9, *P* < 0.001). In Hungary, mosquito abundance was significantly higher in 2023 than in 2024 (*χ*^2^_1_ = 8.02, *P* = 0.007) and higher in spring than in autumn (*χ*^2^_1_ = 19.2, *P* < 0.001). In Spain, mosquito numbers were consistently low and showed no significant variation by year (*χ*^2^_1_ = 0.69, *P* = 0.42) or season (*χ*^2^_1_ = 1.89, *P* = 0.18). Across both countries, pig and sheep farms yielded significantly fewer mosquitoes than cattle farms (pigs: *t* = 4.85, *P* < 0.001; sheep: *t* = 4.87, *P* < 0.001) (see Additional file 2). The total number of female mosquitoes collected was significantly higher than that of males (*χ*^2^_1_ = 34.8, *P* < 0.001). The Mosquito Magnet trap collected significantly more mosquitoes than the BG-Pro trap (1137 versus 295 individuals, respectively, *χ*^2^_1_ = 19.7, *P* < 0.001) (Fig. 4).

Only female mosquitoes were identified to the species level due to their epidemiological relevance as potential pathogen vectors. In Spain, all 107 collected females were analyzed. In Hungary, 592 out of 1275 females (46%) were selected for molecular identification on the basis of farm, season, livestock type, and trapping method to reflect ecological variability.

**Fig. 5:**
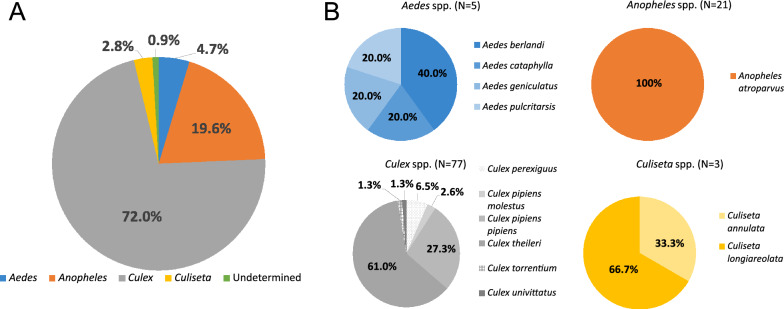
Genera (**A**) and species (**B**) of female mosquitoes collected in Spain during three collections in 2023 and 2024

**Fig. 6:**
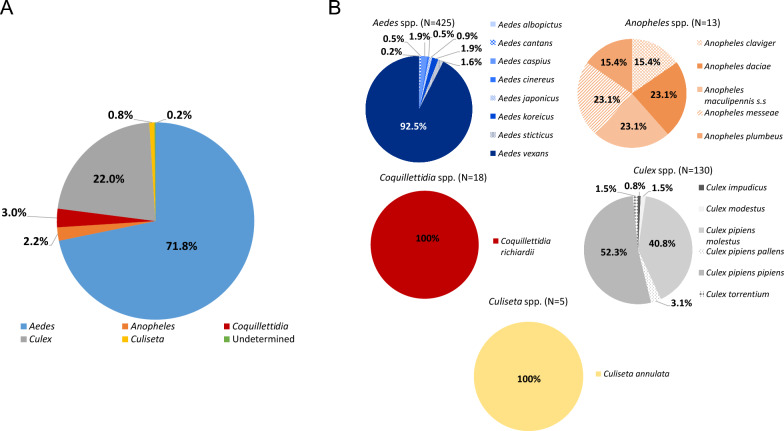
Genera (**A**) and species (**B**) of female mosquitoes collected in Hungary during three collections in 2023 and 2024

Species richness and abundance varied between countries, farms, and host types (Figs. 4, 5, 6; Additional file 2). Overall diversity and abundance were substantially higher in Hungary (*n* = 21 species) than in Spain (*n* = 13 species) (Figs. 5 and 6). In Spain, four genera were identified: six *Culex* species (72%), one *Anopheles* species (19.6%), four *Aedes* species (4.7%), and two *Culiseta* species (2.8%), with one specimen remaining unidentified (Fig. 5). The most abundant species were *Culex theileri* (*n* = 47) and *Cx. pipiens pipiens* (*n* = 21), both present on all farms. In Hungary, five genera were recorded: eight *Aedes* species (71.8%), six *Culex* species (22%), one *Coquilletidia* species (3%; *Cq. richiardii*), five *Anopheles* species (2.2%), and one *Culiseta* species (0.8%; *Cs. annulata*), with one unidentified specimen. (Fig. 6). The most abundant species was *Ae. vexans* (*n* = 393), followed by *Cx. pipiens pipiens* (*n* = 68).

Species composition showed clear geographical segregation between Spain and Hungary. *Aedes* and *Anopheles* assemblages were entirely distinct between countries: all *Aedes* species identified in Spain were absent from Hungary, and the five *Anopheles* species identified in Hungary were absent from Spain, where only *An. atroparvus* was recorded. In contrast, several *Culex* and *Culiseta* species were shared between countries, including *Cx. pipiens molestus, Cx. pipiens pipiens, Cx. torrentium*, and *Cs. annulata* (Additional file 2). *Culex pipiens pipiens* was significantly more abundant in Hungary (*n* = 68), compared with Spain (*n* = 21) (*χ*^2^_1_ = 11.4, *P* = 0.003), and more frequently collected in spring than autumn (*χ*^2^_1_ = 8.6, *P* = 0.008). In Hungarian sites, the *Culex pipiens* complex was widespread, with *Cx. pipiens molestus* particularly abundant, whereas it was rare in Spain. Some *Culex* species were occasionally detected, including *Cx. perexiguus* (*n* = 5), *Cx. univittatus* (*n* = 1), and *Cx. torrentium* (*n* = 1) in Spain, and *Cx. pipiens pallens* (*n* = 4), *Cx. modestus* (*n* = 2), and *Cx. torrentium* (*n* = 2) in Hungary (Additional file 2). *Culiseta* specimens were scarce, with only three and five individuals in Spain and Hungary, respectively. Host-related differences were also observed. In Hungary, the cattle farm (farm 5) showed the highest abundance (*n* = 372) and richness (15 species), followed by the pig farm (farm 6, *n* = 128) and the sheep farm (farm 7, *n* = 91). In Spain, mosquito abundance remained low across all farms, although the mixed sheep-cattle farm (farm 4) showed slightly higher richness (10 species) (Additional file 2).

### Tick collections and identifications

**Fig. 7:**
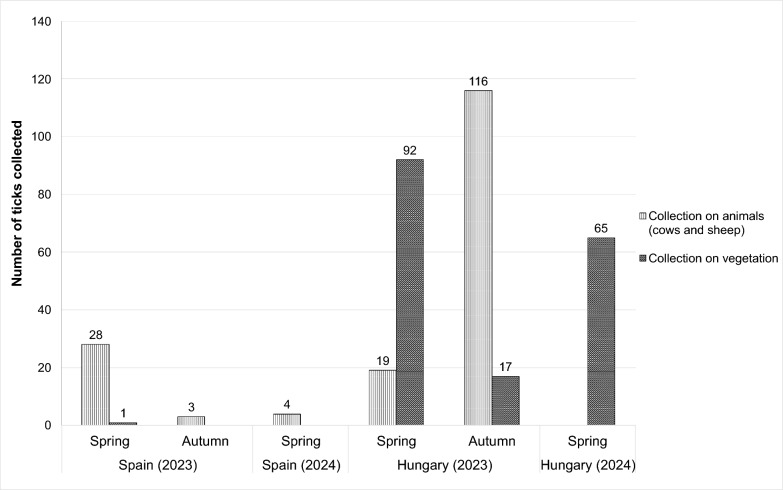
Annual and seasonal variations in number of ticks collected either on animals or on vegetation in Spain and Hungary in 2023 and 2024. Histogram patterns: vertical lines, collection on animals (cows and sheep); grid, collection on vegetation

A total of 345 ixodid ticks were collected in Spain and Hungary, including 155 nymphs, 101 adult females, and 89 adult males (Table 1). Tick abundance differed markedly between countries, with 36 ticks collected in Spain compared with 309 in Hungary (Fig. 7). In Spain, most ticks were collected during spring 2023 (*n* = 29), whereas only three and four ticks were recorded in autumn 2023 and spring 2024, respectively; the lower number in spring 2024 may be explained by the reduced number of cattle examined (five instead of ten). In Spain, all ticks, except one from captured from the vegetation in spring 2023, were collected from animals (15 from sheep and 20 from cattle). In Hungary, sampling was more evenly distributed between vegetation (*n* = 174; 56.3%) and animals (*n* = 135; 43.7%). Among animal-derived ticks, 131 (97%) were collected from cattle and 4 (3%) from sheep. However, it should be noted here that the number of ticks collected from animals in Hungary was probably negatively influenced by the fact that the sheep were treated with ivermectin 1 week prior to both spring sampling sessions, and cattle were housed indoors during spring 2024, limiting tick exposure and resulting in 19 and 0 ticks collected on animals in spring 2023 and 2024, respectively.

**Table 1:** Total number of species and developmental stage of ticks collected according to sampling area in four farms in Spain and three farms in Hungary in 2023 and 2024

		Sampling sites						
		Spain				Hungary		
		1	2	3	4	5	6	7
		Cattle	Pigs	Sheep	Cattle/sheep	Cattle	Pigs	Sheep
*D. marginatus*	Males	0	0	0	0	1 + 0 (spring)*; 43 (autumn)	0	0
	Females	0	0	0	0	2 + 0 (spring)*; 16 (autumn)	0	0
*D. reticulatus*	Nymphs	0	0	0	0	1 (autumn)	0	0
	Males	0	0	0	0	7 (autumn)	0	0
	Females	0	0	0	0	7 (autumn)	0	0
*Hae. concinna*	Nymphs	0	0	0	0	18 + 4 (spring)*; 1 (autumn)	6 + 8 (spring)*; 1 (autumn)	13 + 29 (spring)*; 1 (autumn)
	Males	0	0	0	0	3 + 0 (spring)*	0	1 + 0 (spring)*
	Females	0	0	0	0	21 + 0 (spring)*	0 + 3 (spring)*	2 + 0 (spring)*
*H. lusitanicum*	Males	0	0	2 + 0 (spring)*	8 + 2 (spring)*; 3 (autumn)	0	0	0
	Females	0	0	0	4 + 2 (spring)	0	0	0
*I. ricinus*	Nymphs	0	0	0	0	6 + 6 (spring)*; 1 (autumn)	0	36 + 15 (spring)*; 9 (autumn)
	Males	0	0	0	0	1 + 0 (spring)*; 10 (autumn)	0	0
	Females	0	0	0	0	34 (autumn)	0	1 + 0 (spring)*; 2 (autumn)
*R. bursa*	Males	0	0	7 + 0 (spring)*	1 + 0 (spring)*	0	0	0
	Females	0	0	6 + 0 (spring)*	1 + 0 (spring)*	0	0	0
*Subtotal*		0	0	15 + 0 (spring)*	14 + 4 (spring)*; 3 (autumn)	52 + 10 (spring)*; 120 (autumn)	6 + 11 (spring)*; 1 (autumn)	53 + 44 (spring)*; 12 (autumn)
*Total*		36	309					

Spain, farm 1: Casa de Riosequillo, farm 2: La Bogoña, farm 3: El Carril, farm 4: La Carballa; Hungary, farm 5: Geum Kft, farm 6: Istvándi és Társai Kft, and farm 7: Dörögdi mező Kft. Spring: May and July 2023/2024, autumn: September and October 2023. *D., Dermacentor; Hae., Haemaphysalis; H., Hyalomma; I., Ixodes; R. Rhipicephalus*. *First number corresponds to spring 2023 and second to spring 2024.

**Fig. 8:**
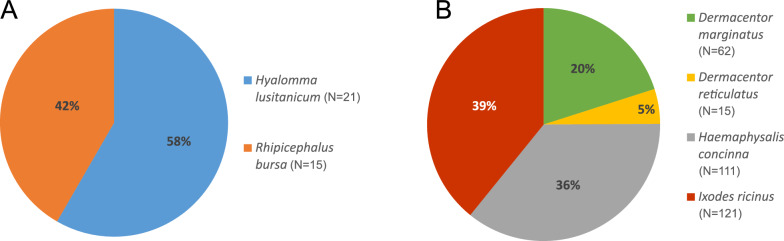
Species of ticks collected in several farms, either on animals or on vegetation, in **A** Spain and **B** Hungary in 2023 and 2024

Tick species composition differed markedly between countries (Fig. 8). In Spain, only two species were recorded: *Hyalomma lusitanicum* (58.3%, *n* = 21) and *Rhipicephalus bursa* (41.7%, *n* = 15). In Hungary, four species were identified: *Ixodes ricinus* (39.2%, *n* = 121), *Haemaphysalis concinna* (35.9%, *n* = 111), *Dermacentor marginatus* (20.1%, *n* = 62), and *D. reticulatus* (4.9%, *n* = 15). *Ixodes ricinus* and *Hae. concinna* were predominantly collected as nymphs, whereas *Dermacentor* spp. were mainly adults. The cattle farm (farm 5) exhibited the highest richness and abundance (four species). The pig farm (farm 6) yielded only *Hae. concinna*, while the sheep farm (farm 7) was dominated by *I. ricinus* and *Hae. concinna* (Table 1). Seasonally, *D. marginatus* and *D. reticulatus* were more frequently recorded in autumn, whereas *I. ricinus* and *Hae. concinna* were detected during in both spring and autumn (Table 1).

### Stable fly collections

**Fig. 9:**
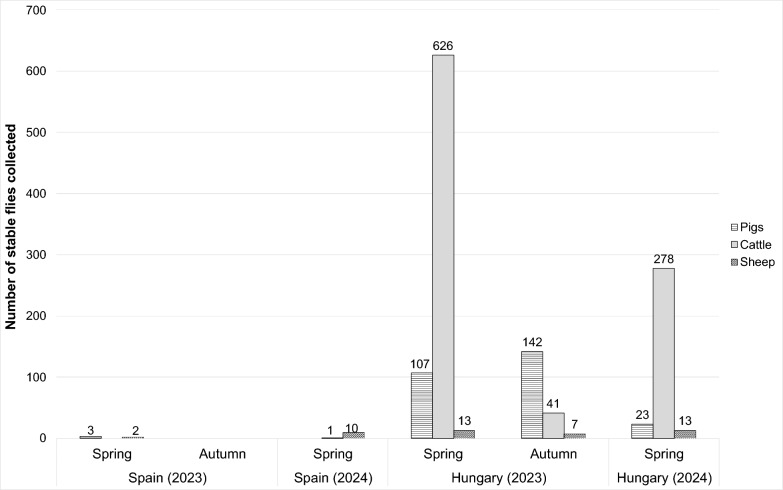
Host-specific collection of stable flies (*Stomoxys calcitrans*) in Spain and in Hungary in 2023 and 2024. Histogram patterns: horizontal lines, pigs; grey rectangles, cattle; diagonal lines, sheep

A total of 1266 stable flies were collected (Table 2; Fig. 9). Abundance differed significantly between countries, with 19 individuals collected in Spain compared with 1250 in Hungary (*χ*^2^_1_ = 11.2, *P* = 0.004), and a sex ratio that varied among farms. All individuals were identified as *Stomoxys calcitrans*.

**Table 2:** Sex distribution of stable flies (*Stomoxys* *calcitrans*) collected at sampling sites in Spain (farms 1–4) and Hungary (farms 5–7)

Farms	Sampling sites						
	Spain				Hungary		
	1 Cattle	2 Pigs	3 Sheep	4 Cattle/sheep	5 Cattle	6 Pigs	7 Sheep
Males	2	1	7	0	855	197	10
Females	1	2	3	0	90	75	23
Total	3	3	10	0	945	272	33

Spain, farm 1: Casa de Riosequillo, farm 2: La Bogoña, farm 3: El Carril, farm 4: La Carballa; Hungary, farm 5: Geum Kft, farm 6: Istvándi és Társai Kft, and farm 7: Dörögdi mező Kft

In Spain, stable fly abundance remained low across all farms, with a maximum of ten individuals recorded at farm 3 (sheep), and fewer than five specimens per farm elsewhere. The limited numbers of collected specimens in this country precluded meaningful comparisons between host types or season, although all individuals collected in spring 2024 were associated with sheep. In total, 5 flies were collected in spring 2023 and 11 in spring 2024, whereas no specimens were recorded in autumn 2023.

In Hungary, the majority of specimens (*n* = 945, 78.6%) were collected at the cattle farm (*χ*^2^_2_ = 13.5, *P* = 0.008), followed by the pig farm (*n* = 272) and sheep farm (*n* = 33). However, pigs accounted for a higher proportion of captures during autumn 2023 (74.7%). Across Hungarian study sites, livestock type was significantly associated with stable fly abundance, with cattle farm yielding significantly higher numbers of flies than sheep farm (*t* = 3.74, *P* = 0.002) or pig farm (*t* = 3.37, *P* = 0.004). Seasonal variation was also observed, with significantly more stable flies collected in spring than in autumn (Fig. 9, *χ*^2^_1_ = 9.95, *P* = 0.007). In Hungary, activity peaked in spring 2023 (*n* = 746 specimens) and remained high in spring 2024 (*n* = 314). Although autumn yielded fewer total captures (*n* = 190), a relatively higher proportion was associated with pig farm (Fig. 9).

## Discussion

Arthropod vectors are particularly sensitive to ongoing global environmental changes across Europe, including climate shifts, land-use modifications, and intensification of agricultural systems. Farms may therefore represent critical yet underexplored hotspots for vector circulation and pathogen transmission. Within extensive farming environments, four major host populations—humans, livestock, companion animals, and wildlife—coexist and interact closely, creating complex ecological interfaces that may facilitate vector-borne disease dynamics. Despite growing recognition of the importance of these agro-ecosystems, relatively few studies have simultaneously examined vector community composition across different livestock systems while also accounting for contrasting climatic contexts. Most available research has focused on a single vector taxon, specific host species, or isolated geographic areas, limiting our understanding of how farming practices and regional climate jointly shape vector assemblages [34]. In this context, our exploratory study sought to characterize vector community occurrence on farms hosting three distinct livestock species—cattle, pigs, and sheep—in two European countries with markedly different climatic regimes: Spain and Hungary. We present evidence that studied arthropod species composition and/or density differed markedly between countries and livestock farms.

The high mosquito diversity observed in Hungary aligns with previous surveys reporting floodplain-associated taxa, particularly *Ae. vexans* and the *Cx. pipiens* complex [35, 36]. Their ecological adaptability and close association with livestock settings, combined with the presence of wetlands, river systems, and irrigated areas, likely promote large populations of generalist species [37]. Farm-level mosquito assemblages largely reflected local hydrology and management practices. The cattle farm showed the highest richness and abundance, dominated by *Ae. vexans* and *Cx. pipiens*, likely supported by permanent water bodies, irrigation systems, and manure-rich microhabitats [38]. In contrast, the pig and sheep farms hosted fewer individuals but a more diverse composition, with container-breeding invasive species such as *Ae. koreicus* and *Ae. japonicus* detected at the pig facility—matching their preference for shaded, peri-domestic environments [38]. Within the *Cx. pipiens* complex, *Cx. pipiens pipiens*, *Cx. pipiens molestus*, and *Cx. pipiens pallens* were identified, illustrating the ecological and phenotypic plasticity of the members of this complex [39, 40]. The *Cx. pipiens* complex is regarded as a major European vector taxa, particularly of WNV, which has increasingly been reported in Europe over the past decades [9]. *Culex torrentium* and *Cx. modestus* were collected at the cattle and sheep farms, respectively, consistent with their known geographic distribution [41, 42]. Finally, the detection of *Cx. impudicus* at the cattle farm in Somogy county, despite limited previous records, may reflect localized establishment, underreporting, or earlier misidentifications, as suggested by recent national updates [36, 43]. Invasive *Aedes albopictus*, *Ae. koreicus,*, and *Ae. japonicus* are now established across Central Europe [39]. *Aedes albopictus*, collected at the cattle farm, is one of the most invasive mosquito species worldwide and a major vector of arboviruses of public health concern including dengue, Chikungunya. and Zika viruses. It has already colonized large parts of Hungary, including the counties investigated in this study. *Aedes japonicus*, detected at both cattle and pig farms, is now widely established throughout the country, including the southwestern regions where our sampling farms are located. The presence of *Ae. koreicus* on the pig farm is consistent with previous reports from Veszprém County (central-western Hungary), where the species is already established. Its absence from Somogy County, in southern Hungary where the cattle farm is located, aligns with the lack of previous records from this area. Five *Anopheles* species were identified (*An. claviger, An. daciae, An. maculipennis, An. messeae*, and *An. plumbeus*), also consistent with their known distribution in Hungary [36]. Among them, *An. maculipennis* and *An. messeae* are of particular epidemiological importance, as they are recognized vectors of *Plasmodium vivax* malaria in Europe [44, 45].

In Spain, farm 4 (cattle/sheep) was the most productive site for mosquitoes, dominated by *Cx. theileri* and *Cx. pipiens pipiens*, species well adapted to residual pools and livestock troughs typical of semi-arid pastures [38, 46]. Farms 1–3 (cattle, pig, sheep) yielded fewer mosquitoes overall; however, the presence of *Cx. theileri*, *Cx. perexiguus*, and *An. atroparvus* was consistent with scattered water points and small reservoirs surrounding the farms. The detection of *An. atroparvus* at the pig farm, despite the relatively low number of specimens collected (*n* = 15), may be explained by piglet nurseries with watering areas that provide suitable larval habitats and shelters for the adults [46, 47]. In contrast, the sheep farm yielded a higher number of mosquitoes (*n* = 41), likely due to the presence of permanent water sources containing organic matter and creating stable microhabitats favorable for larval development and adult activity. Overall, these findings indicate that, even with limited rainfall, small-scale hydrological management (e.g., watering frequency, trough maintenance, temporary channel closure) can substantially influence mosquito productivity in Mediterranean livestock systems [46].

Four tick species were identified in Hungary: *I. ricinus*, *Hae. concinna*, *D. marginatus*, and *D. reticulatus*, consistent with previous national records and findings from Central and Eastern Europe, where wooded, humid landscapes and mixed agricultural systems provide favorable conditions for these questing species [20, 48]. In agreement with the known seasonal activity patterns of these tick species in Europe, a higher number of ticks was collected from vegetation in spring compared with autumn. In contrast, the lower number of ticks collected from animals during the two spring surveys may be attributed to treatments administered prior to sampling. The cattle farm showed the highest species richness and abundance, likely reflecting greater host biomass, open pasture structure, and humid microhabitats around manure and shaded areas that enhance off-host survival and questing success [49]. At the pig farm, where only *Hae. concinna* was collected, the triple African swine fever (ASF) fencing system is most likely reducing wildlife–livestock interactions and limited the introduction of additional tick species. The dominance of *I. ricinus* and *Hae. concinna* on sheep farms is consistent with extensive grazing near forest margins, characterized by shaded leaf litter and higher ground-level humidity [23, 50]. Nymphs represented the majority of *I. ricinus* and *Hae. concinna* specimens, whereas *D. marginatus* and *D. reticulatus* were almost exclusively collected as adults. This pattern is expected as their immature stages are primarily endophilic, feeding on small mammals, and therefore rarely collected by flagging or on large mammals. The most prevalent species, *I. ricinus*, was mainly recorded on cattle and sheep farms. It is the most important tick vector in Europe for both human and animal health, transmitting a wide range of pathogens, including bacteria such as *Borrelia burgdorferi* s.l. and *Anaplasma* spp., parasites such as *Babesia* spp., or viruses such as tick-borne encephalitis virus (TBE) or Louping ill virus [23]. *Haemaphysalis concinna*, although less extensively studied, has been increasingly reported in Hungary, possibly in relation to ecological and climatic changes, as it thrives in warm-temperate and moist environments, and is implicated in the transmission of *Rickettsia* and *Babesia* species [50]. In our study, it was particularly associated with pigs and sheep, in line with its affinity for medium-sized mammals [50]. Adults of *D. marginatus* and *D. reticulatus* were restricted to cattle farm, consistent with their preference for large mammalian hosts at the adult stage [51]. Both species are recognized as vectors of several pathogens, including *Babesia canis* and *Rickettsia raoultii* for *D. reticulatus*, and *Rickettsia slovaca* or *R. raoultii* for *D. marginatus* [51]. Their current distribution and expansion in Central Europe has been linked to climate change and land-use modifications, particularly in agricultural landscapes [52].

Consistent with the lower tick diversity typically observed in Mediterranean environments compared with temperate humid regions, due to arid climatic conditions, host availability, and habitat structure [53], tick diversity and abundance were lower in Spain than in Hungary. Only *H. lusitanicum* and *R. bursa* were identified, and exclusively at the sheep farm (El Carril) and the mixed cattle/sheep farm (La Carballa). In agreement with its ecology, *H. lusitanicum*, historically dominant in central and southern Spain and well adapted to dry, open habitats, was mainly collected during spring surveys [54]. Its ditropic life cycle—endophilic immature stages feeding primarily on lagomorphs and adults on ungulates—together with a mixed host-seeking strategy, likely explains why only adults were collected on livestock. Moreover, in Mediterranean climates, the sampling periods may not coincide with the emergence or host-seeking activity of juvenile stages [54]. Although traditionally considered of limited public health relevance, human infestations by *H. lusitanicum* are increasingly reported in rural Spain [54]. Importantly, it is a vector of several pathogens affecting wildlife and livestock, including *Theileria* spp. and *Coxiella burnetii*, and is regarded as the principal vector of Crimean-Congo hemorrhagic fever virus (CCHFV) in the Iberian Peninsula [54, 55]. *Rhipicephalus bursa*, a two-host questing tick characteristic of Mediterranean environments, infest small ruminants such as sheep and goats, as well as cattle and horses. It has been associated with the transmission of multiple pathogens, including *Anaplasma marginale, Babesia bigemina*, and *B. bovis* in cattle; *A. ovis, B. motasi, B. ovis, Ehrlichia ovina*, and *Theileria separata* in sheep; and *Babesia caballi* and *Theileria equi* in horses. It is also suspected to be implicated in the transmission of CCHFV [56, 57].

Almost all stable flies (98.7%) were collected in Hungary, during the spring seasons, and all were identified as *Stomoxys calcitrans*. The cattle farm accounted for approximately 80% of captures and showed a strong male bias (90.5%). This pattern is consistent with known ecological features of *S. calcitrans*: (i) abundant hosts and sun-exposed trap placement near manure heaps—optimal larval substrates—favor high local production [58]; (ii) sex-specific responses to olfactory and visual cues, as well as seasonal ecological factors, may lead to male-biased trap catches, suggesting a possible sampling effect rather than true population structure [21, 59]; and (iii) spring population peaks are typical in temperate climates [60]. At the pig farm, relatively high catches were consistent with outdoor access areas, muddy substrates, and building edges that provide suitable resting and developmental sites. The sheep farm yielded comparatively few individuals, likely due to lower host biomass and fewer nutrient-rich larval substrates than at the cattle farm [58]. Reports of extreme abundances elsewhere—for example, nearly 20,000 individuals trapped during late-summer peaks in southwest England [61] and other parts of Central Europe [21]—highlight the strong influence of local environmental conditions and host availability on stable fly dynamics. Beyond their nuisance and economic impact, stable flies are recognized mechanical vectors of several veterinary pathogens, including *Besnoitia besnoiti*, lumpy skin disease virus, and African swine fever virus [7, 62, 63]. Their capacity for dispersal over several kilometers further enhances their epidemiological relevance [64].

*Stomoxys calcitrans* were found at very low densities in Spain, likely due to high temperatures and low humidity, limiting larval survival and adult activity. Although the species is widely distributed across the country, its abundance strongly depends on microclimatic conditions, farm management, and substrate moisture [58]. Our low capture rates are consistent with the hot, dry conditions prevailing during the sampling period. Overall, microclimatic constraints and limited availability of suitable breeding substrates probably maintained populations below detectable levels despite the species’ broad national distribution [58].

When comparing the two studied countries, almost 10 times more mosquitoes and ticks, and nearly 80 times more stable flies, were collected in Hungary than in Spain. Species diversity was also higher in Hungary for both mosquitoes and ticks. These results are consistent with previous studies reporting greater vector densities in Central and Eastern Europe compared with drier Mediterranean regions [20, 37]. Mosquito species richness was likewise higher in Hungary, supporting the idea that mosquito assemblages in Central Europe are primarily shaped by water availability, floodplains, irrigation canals, and stable freshwater habitats that provide diverse larval breeding sites in continental environments. In contrast, Mediterranean ecosystems tend to constrain mosquito diversity and favor species adapted to drier conditions [35–37]. In both countries, at the local scale, cattle farms supported higher mosquito abundances than pig or sheep farms. This pattern likely reflects the larger host body size of cattle and the more consistent availability of water from irrigation systems or drinking facilities. These findings suggest that mosquito productivity is shaped by broader ecological conditions and may be locally enhanced in livestock systems, particularly in cattle-dominated systems [22, 35]. For ticks, humidity and temperature, both closely linked to climate, were the main explanatory factors for species distribution at the country level, with Mediterranean species predominating in Spain and temperate species in Hungary [52, 53]. At the local scale, farm-specific factors, including environmental characteristics, the presence and diversity of wild fauna and livestock, and farming practices (e.g., African swine fever biosecurity regulations requiring triple fencing, ivermectin treatments, or indoor housing of animals, as observed in the present study), played a key role in shaping tick community composition and abundance [65]. Such farm-level effects were also evident for stable flies, as manure heaps provide essential breeding substrates and can substantially increase local *Stomoxys calcitrans* densities [58].

Finally, the overall arthropod diversity recorded across the two surveyed countries comprised 37 species, including 30 mosquito species, 6 tick species, and *S. calcitrans*. Among these taxa, 23 species are recognized vectors of pathogens, representing a substantial proportion of the collected entomological fauna. These included 16 mosquito species, all 6 tick species, and *S. calcitrans*, highlighting the epidemiological relevance of the sampled communities. Taken together, our results showed that, in both Spain and Hungary, the livestock farms investigated in this study constitute biodiversity hotspots in terms of arthropods, including vectors that may potentially impact both human and animal health. Our study has several limitations that must be taken into account when interpreting the results, particularly with regard to the sampling frequency, as only three collections sessions were conducted in each country. On the basis of the findings presented here, several steps should be considered for a more comprehensive future study. First, additional surveys conducted over multiple years and seasons would be valuable to consolidate the present results and provide a more comprehensive picture of vector community dynamics, including both interannual and seasonal variability in livestock farms. Second, the inclusion of other vectors of major veterinary importance, such as *Culicoides* biting midges (notably implicated in bluetongue virus transmission), should be considered in future research to allow for a more complete assessment of vector diversity. Third, increasing the number of farms and livestock systems investigated would be necessary to validate the preliminary trends identified in the present study regarding the influence of animal species and farm environment on vector density and vector-borne pathogen prevalence and diversity. Fourth, broader sampling would also be required to assess the actual impact of certain livestock management practices observed on some farms (see Additional File 1), such as the use of antiparasitic treatments (e.g., ivermectin), insecticide or repellent applications, or the housing of animals indoors. However, these effects are likely short-lived and cannot fully explain the broader contrasts observed between Spain and Hungary. Finally, conducting fieldwork in livestock settings posed challenges for tick collection directly from animals. Beef cattle breeds and Iberian pigs reared for “Pata Negra” ham production were particularly difficult to handle. Although sedation could allow for more exhaustive and safer tick inspections, it remains impractical in most farm contexts.

## Conclusions

The differences highlighted between the farms studied in Spain and Hungary, representing contrasting context, support the view that vector community structure is shaped by the interplay among climate, habitat, livestock type, livestock management practices, and seasonality. These findings underscore the importance of integrated vector surveillance within a One Health framework to anticipate emerging risks. From an epidemiological perspective, the occurrence of 23 vector species in both countries, including major vectors such as *Cx. pipiens pipiens*, *Ae. vexans*, *H. lusitanicum*, *I. ricinus*, and *S. calcitrans*, highlights the potential risk of circulation of zoonotic and veterinary pathogens, including WNV, *B. burgdorferi*, *Babesia* spp., or CCHFV. The presence of four members of the *An*. *maculipennis* subgroup further underlines the potential epidemiological role of Hungarian farms as reservoirs of species historically involved in malaria transmission. Climate change is expected to further reshape the risk of VBDs, facilitating the northward and altitudinal expansion of several tick species, while also modifying mosquito and stable fly habitats and diversity with invasive species. Therefore, integrating ecological, climatic, and management perspectives will be crucial to inform effective surveillance and control strategies for VBDs in European livestock systems under conditions of ongoing global change.

## Supplementary Information

Additional file 1.

Additional file 2.

## Data Availability

All data supporting the conclusions of this article are included within the article and supporting materials.
